# Various Sizes and Shapes of Mixed-Anion Fe(NH_2_trz)_3_(BF_4_)_2−x_(SiF_6_)_x/2_@SiO_2_ Nanohybrid Particles Undergoing Spin Crossover Just Above Room Temperature

**DOI:** 10.3390/nano15020090

**Published:** 2025-01-09

**Authors:** Xinyu Yang, Rafal Bielas, Vincent Collière, Lionel Salmon, Azzedine Bousseksou

**Affiliations:** Laboratoire de Chimie de Coordination, CNRS & Université de Toulouse (UPS, INP), 31077 Toulouse, France

**Keywords:** spin crossover, iron(II), triazole, reverse micelles, nanoparticles

## Abstract

Spin crossover (SCO) iron (II) coordination compounds in the form of nanohybrid SCO@SiO_2_ particles were prepared using a reverse micelles technique based on the TritonX-100/cyclohexane/water ternary system. Tetraethyl orthosilicate (TEOS) acts as precursor of both the SiF_6_^2−^ counter-anion and SiO_2_ to obtain Fe(NH_2_trz)_3_(BF_4_)_2−x_(SiF_6_)_x/2_@SiO_2_ nanoparticles with different sizes and morphologies while modifying the TEOS concentration and reaction time. The adjustable mixed-anion strategy leads to a range of quite scarce abrupt spin crossover behaviors with hysteresis just above room temperature (ca. 293 K), which is very promising for the integration of these materials into functional devices.

## 1. Introduction

Spin crossover (SCO) complexes are fascinating switchable materials known notably for their variable magnetic and optical properties in response to external stimuli, such as temperature variation, making them highly promising candidates for use in photonic, electronic, and mechanical devices [1,2,3]. Despite numerous reports documenting SCO behavior across a temperature range of 80 to 500 K in various multi-dimensional metal complexes [4,5,6,7,8,9,10,11], there is an ongoing search for stable, integrable compounds with transition temperatures just above room temperature. This search is particularly focused on materials that exhibit a hysteresis loop, providing a memory effect for functional applications. Among the myriad of metal complexes studied for SCO properties, iron(II) compounds stand out, particularly those from the Fe-triazole coordination polymers family. These compounds are notable for forming one-dimensional chains of iron ions, each bridged by three neutral triazole ligands. This unique structural configuration facilitates robust cooperative interactions within the chains, significantly influencing their SCO characteristics [12,13]. Controlling the spin state in metal complexes remains a significant challenge, largely due to the intricate and often unpredictable nature of the secondary coordination sphere. This sphere plays a critical role in determining the structural properties and, consequently, the SCO behavior of these complexes. Accurate prediction and manipulation of these structural properties are essential for advancing the application of SCO materials, yet they present considerable difficulties. The general approaches to controlling the spin crossover behavior (transition temperatures, abruptness, hysteresis loop width) are based on intrinsic chemical modification or the guest effect, the particle size effect, or the extrinsic matrix effect [14,15,16,17,18,19,20,21,22]. One effective strategy to modify the spin transition temperature in this family of compounds is through partial substitution of either the ligand or the metal within the coordination complexes [23,24,25,26,27,28,29,30]. Typically, this process involves mixing the corresponding precursors in precise ratios to modulate the final chemical composition of the product. Despite its potential, synthesizing alternative pure mixed counter-anion derivatives has proven to be particularly challenging. Previous attempts have often resulted in physical mixtures rather than true mixed counter-anion complexes, with no clear results reported thus far [31,32]. Recently, we succeeded in synthesizing a pure mixed counter-anion Fe(NH_2_trz)_3_(BF_4_)_2−x_(SiF_6_)_x/2_ complex using both the conventional coordination chemistry method (mixing precursors) or post synthetic modification approaches using tetraethyl orthosilicate (TEOS) as the SiF_6_^2−^ precursor, showing just above room temperature spin transition with a narrow hysteresis loop [33,34]. Nevertheless, control of transition temperatures and morphology of the particles were not possible at the same time. Here, we show that it can be done using a reverse micelles technique with the TritonX-100/cyclohexane/water ternary system [28,35,36,37,38]. In this series of experiments, TEOS acts as precursor of both the SiF_6_^2−^ counter-anion and the SiO_2_ to obtain functionalizable Fe(NH_2_trz)_3_(BF_4_)_2−x_(SiF_6_)_x/2_@SiO_2_ nanohybrid particles with different sizes and morphologies, modifying the TEOS concentration and reaction time. This synthetic approach reinforces the tendency to develop advanced spin crossover nano-objects for their integration into functional devices [3,39,40,41].

## 2. Experimental Section

### 2.1. Synthesis of the Spin Crossover Complex Nanoparticles

All reagents were purchased from Sigma Aldrich (Saint-Louis, MO, USA) and used without further purification. Synthesis of Fe(NH_2_trz)_3_(BF_4_)_2−x_(SiF_6_)_x/2_@SiO_2_ nanoparticles (**1**–**6**) consists of preparing two independent micro-emulsions containing the iron salt and the ligand, respectively, and mixing them [28]. For sample **1**, an aqueous solution of Fe(BF_4_)_2_·6H_2_O (373 mg in 1 mL of H_2_O) was added drop by drop and under vigorous agitation to a mixture of Triton X-100 (1.8 mL), hexanol (1.8 mL), cyclohexane (7.5 mL), and TEOS (25 μL) (microemulsion 1). An aqueous solution of NH_2_-triazole (252 mg in 1 mL of H_2_O) was added drop by drop and under vigorous agitation to a mixture Triton X-100 (1.8 mL), hexanol (1.8 mL), cyclohexane (7.5 mL), and TEOS (25 μL) (microemulsion 2). After 15 min, the two clear micro-emulsions were quickly combined and stirred overnight (15 h) at room temperature. The mixture was washed with ethanol and diethyl ether, and nanoparticles were collected through centrifugation at 4000 rpm over 5 min. The series of Fe(NH_2_trz)_3_(BF_4_)_2−x_(SiF_6_)_x/2_@SiO_2_ nanoparticles (**1**–**6**) was obtained with a similar procedure to sample **1** while modifying the quantity of TEOS from 0.25 to 1 equivalents vs. iron salt and the reaction time overnight to 7 days, according to Table 1. Thermogravimetry analysis (TGA) revealed the inclusion of 0.5–0.8 molecules of water and the thermal stability of the complex up to ca. 230 °C for all complexes (see Figure S1). Infrared spectroscopy showed the characteristic vibration modes of the two counter-anions (Figure S2). An intense band attributed to the BF_4_^−^ anions is observed at 1020 and 1095 cm^−1^, while the ν3 stretching vibration band at 730 cm^−1^ and the ν4 bending vibration band at 470 cm^−1^ are features of the SiF_6_^2−^ ion [42].

### 2.2. Sample Characterization

Thermogravimetric analyses were conducted in an inert nitrogen atmosphere using a Mettler Toledo (Columbus, OH, USA) 3+ thermal analyser. Fourier-transform infrared spectroscopy (FTIR) spectra were recorded at room temperature with a PerkinElmer (Waltham, MA, USA) Spectrum 100 spectrometer in ATR mode (resolution ca. 1 cm^−1^) between 650 cm^−1^ and 3500 cm^−1^. Nuclear magnetic resonance (NMR) experiments were recorded on a Bruker Avance 400 III HD spectrometer operating at magnetic fields of 9.4 T. Samples were packed into a 3.2 mm rotor and spun at different spinning rates (typically between 9 to 18 kHz) at a low temperature (external temperature of 231 K). ^19^F magical angle spinning (MAS) solid-state nuclear magnetic resonance (ssNMR) was measured with a Hahn-echo scheme synchronized with the spinning rate and a relaxation delay of 10 s. ^29^Si CP (CP = Cross-Polarization) MAS spectra were recorded with a recycle delay of 1.5 s and a contact time of 3 ms. ^29^Si MAS were acquired with single pulse experiments with recycle delays of 3 s. Chemical shifts were externally referenced to CCl_3_F and tetramethylsilane for ^19^F and ^29^Si, respectively. Powder X-ray diffraction patterns were recorded using a PANalytical (Malvern, UK) X’Pert equipped with a Cu X-ray tube, a Ge(111) incident beam monochromator (λ = 1.5406 Å), and an X’Celerator detector. The size and morphology of the SCO particles were determined through transmission electron microscopy (TEM) using a JEOL (Peabody, MA, USA) JEM-1011 (accelerating voltage 100 kV). TEM samples were prepared by placing a drop of the particles (suspended in ethanol) on a carbon-coated copper grid. High-resolution Scanning Transmission Electron Microscopy High Angle Annular Dark-Field (HRSTEM-HAADF) images and Energy Dispersive X-ray Spectroscopy (EDS) analyses were recorded using a JEOL (Peabody, MA, USA) TEM JEM ARM200F Cold FEG (accelerating voltage of 200 kV) equipped with an EDX spectrometer. Variable-temperature optical reflectivity data were acquired with a MOTIC (Wetzlar, Germany) SMZ-168 stereomicroscope equipped with MOTICAM 1000 color CMOS camera. A 2 K min^−1^ rate was used for both cooling and heating.

## 3. Results

**Figure 1:**
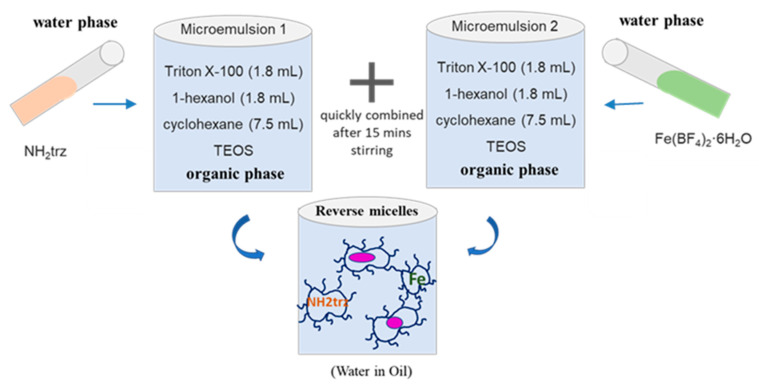
Schematic representation of the reverse micelles method.

Based on already reported results [28], reverse microemulsions with Triton X-100 as the surfactant were used to obtain a series of functionalized particles. Figure 1 shows the schematic representation of the reverse micelles method used to synthesize the different batches of particles. The use of such a microemulsion permits us to confine seed particles and to limit and homogenize their growth [43,44,45,46,47]. In order to obtain pure particle samples of the mixed-anions Fe(NH_2_trz)_3_(BF_4_)_2−x_(SiF_6_)_x/2_ complex, a controlled quantity of TEOS was used, and the reactions were performed in a plastic container to avoid competition with the SiO_2_ ripped from the wall of the glass container [33,34]. In fact, SiO_2_ can react with HF formed through the hydrolysis of the BF_4_^−^ anions to form SiF_6_^2−^ anions. It is interesting to note that during the formation of the similar core@shell [Fe(Htrz)_2_(trz)](BF_4_)@SiO_2_ nanoparticles in a glass container, the BF_4_^−^ anion was not exchanged by SiF_6_^2−^, underscoring the critical role of the NH_2_trz ligand in the anion substitution mechanism [28,29].

From our previous results, we already know that the experimental conditions for reverse micelles synthesis affect the spin crossover properties and morphology of the resulting [Fe(Htrz)_2_(trz)](BF_4_)@SiO_2_ nanoparticles [38]. As shown in Figure 1, the TEOS silica precursor was added to the organic phase (cyclohexane) containing hexanol as a co-surfactant. In a series of experiments, the quantity of TEOS (ranging from 0.25 to 1 eq in the organic phase) was varied while keeping other parameters constant, like ω (the surfactant/water ratio). In addition, the effect of the reaction time (15 h or 7 days) was also investigated. Table 1 summarizes the experimental conditions for the different nanoparticle syntheses.

**Figure 2:**
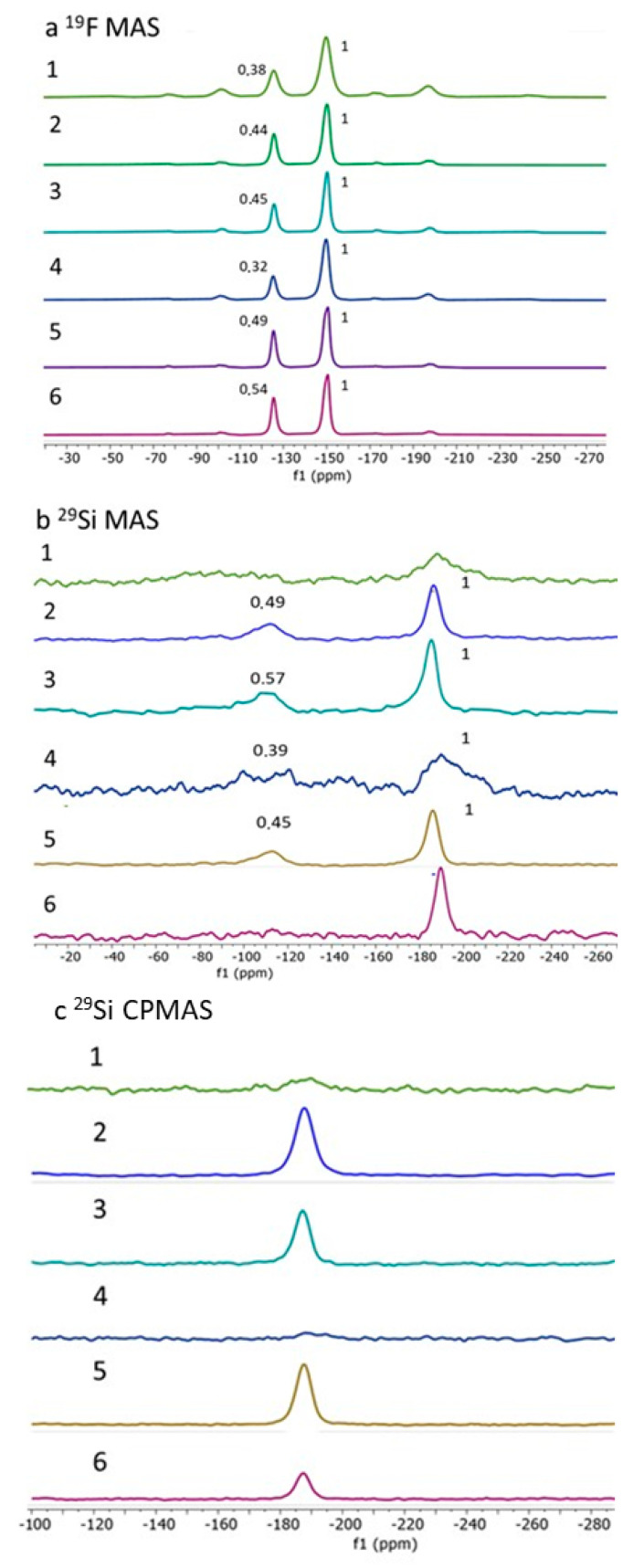
Solid-state NMR: (**a**) ^19^F MAS with ν_r_ 18 kHz; (**b**) ^29^Si MAS with ν_r_ 8 kHz; (**c**) ^29^Si CPMAS with ν_r_ 8 kHz; (**c**) spectra obtained at 232 K for sample **1**–**6**.

As already reported, the content of SiO_2_ is rather challenging to determine through elemental analysis, and solid-state NMR appears to be more suitable for comparing the SiF_6_^2−^/BF_4_^−^ ratio and to give a tentative composition of the different samples [34]. The resolution of the SSNMR spectra can be greatly improved by using the magic angle spinning technique (MAS). However, MAS spinning results in a temperature increase of the sample because of the friction between the air and the rotating rotor, which switch the sample in the paramagnetic high-spin state. Thus, measurements have to be performed at a low temperature to keep the iron complex diamagnetic during the NMR acquisition. Figure 2 present the ^19^F Hahn-echo, ^29^Si MAS SSNMR, and ^29^Si CP MAS spectra for the different samples, with spinning frequencies (νr) of 18 kHz and 8 kHz, respectively, at a low temperature of 232 K. The ^19^F MAS Hahn-echo spectra (Figure 2a) clearly display isotropic resonances corresponding to the BF_4_^−^ and SiF_6_^2−^ anions at −150 ppm and −125 ppm, respectively, consistent with previous results [38]. By comparing their integration and the corresponding SiF_6_^2−^/BF_4_^−^ intensity ratios for all samples in this series, which range from 0.32 ± 0.02 to 0.54 ± 0.02, we observe that increasing either the quantity of TEOS or the reaction time leads to a higher content of SiF_6_^2−^.

For all samples, the ^29^Si MAS spectrum exhibits a peak at −188 ppm, which is attributed to the SiF_6_^2−^ anion, as shown in Figure 2b. Additionally, samples **2** (0.5 eq TEOS, 15 h), **3** (1 eq TEOS, 15 h), and **5** (0.5 eq TEOS, 7 d) show a clear peak at −112 ppm, attributed to the Q4 siloxane bridge (SiO)_4_Si(SiO)_4_Si. This observation is compatible with the assumption of a structure with the SCO complex as the core and the SiO_2_ grafted at the surface. In contrast, for samples **1** (0.25 eq TEOS, 15 h) and **4** (0.25 eq TEOS, 7 d), Q4 peaks are difficult to observe because of (1) the insufficient amount of TEOS, making it difficult to form the SiO_2_ grafting and (2) the broadening/shift of the peaks due to a residual paramagnetic fraction for the given experimental conditions in agreement with lower transition temperatures in comparison with other samples. In other words, 232 K is not enough to cool samples **1** and **4** in their fully LS state during the NMR measurement. Surprisingly, for sample **6** (1 eq TEOS, 7 d), the grafting of silica around the nanoparticles is not detected. This will require further study, but it is conceivable that over time the silica is transformed into SiF_6_^2−^ counter-anions. Moreover, the presence of independent SiO_2_ nanoparticles is ruled out by the absence of the Q3 peak in the ^29^Si CPMAS spectra that would appear near −100 ppm. All of the NMR data were combined to propose a chemical composition for all synthesized compounds, which is given in Table 1. To complete the composition analysis, powder X-ray diffraction measurements were performed on selected representative samples **5** and **6** (Figure S3). In agreement with the NMR results, the diffractogram for sample **6** does not show additional peak attributable to the silica like that observed at 22° for the sample **5**. Moreover, although samples are isostructural to similar complexes obtained through other synthetic methods [32,33,34], we observed a broadening of the peaks, which can be attributed to the size effect of the crystallites/particles and/or the difference in crystallinity.

In comparison with the previously used post synthetic modification approach [33,34], the composition appears to be more controllable while increasing the quantity of TEOS from 0.25 to 0.5 eq. Nevertheless, when the reaction time is set to 15 h, increasing the equivalent amount of TEOS to 1 eq does not significantly alter the insertion of SiF_6_^2−^ anions, as observed in samples **2** and **3**. However, by simultaneously increasing both the reaction time and the amount of TEOS, the insertion of SiF_6_^2−^ anions can be further enhanced, as seen in samples **5** and **6**. Naturally, the composition of these compounds also influences their morphology and self-assembly properties, which we will discuss in the following sections.

**Figure 3:**
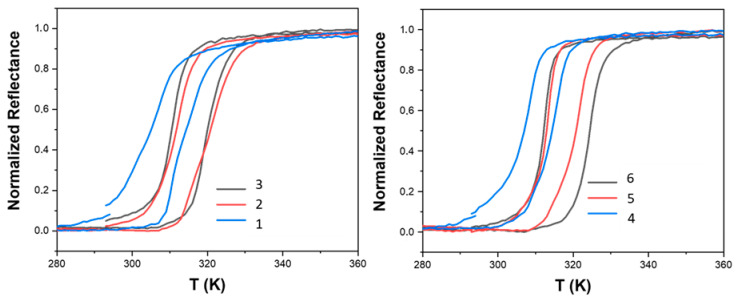
Thermal variation of the optical reflectance (desolvated sample) in the heating and cooling mode for samples **1**–**6**.

The homogeneity and the spin crossover properties of the samples were probed through optical reflectivity measurement. Figure 3 shows the thermal cycle for the desolvated samples **1**–**6** and illustrates the spin crossover properties. Figure S3 gathers the results obtained for consecutive thermal cycles, revealing the well-known initial desolvatation/structural run-in effect on the spin crossover properties. Whatever the experimental conditions, the SCO profile is uniform in comparison with the stepped plots observed for similar compounds obtained through the conventional coordination chemistry method [33,34]. For overnight reactions (Figure 3), increasing the equivalent of TEOS from 0.25 to 0.5 relative to iron results in a noticeable elevation of the transition temperatures, in agreement with the determined compositions.

Table 1 also gathers all of the transition temperatures for all samples. The results suggest that the amount of TEOS plays a significant role in modulating the thermal properties of the complexes, likely by affecting the microenvironment around the iron centers. However, further increasing the TEOS equivalent to 1 does not seem to confer any additional benefit in terms of raising the transition temperatures and composition. This plateau indicates that there might be a saturation point beyond which additional TEOS does not further alter the spin crossover properties, possibly due to the limitation in the incorporation of additional silicate units into the complex structure. Furthermore, the spin transition temperature can be further tuned by extending the reaction time. For example, sample **6**, obtained after a 7-day reaction period, shows a higher transition temperature compared to sample **3**, which was synthesized overnight both with 1 equivalent of TEOS.

The enhancement of the transition temperature with prolonged reaction time highlights the dynamic nature of the complex formation process. This observation aligns with the presence of a mixed-anion composition and the increased incorporation of SiF_6_^2−^ ions. The insertion of these anions likely contributes to the stabilization of the low spin state, thereby raising the spin transition threshold. This relationship between reaction conditions and spin crossover properties is crucial for tailoring the material’s performance for specific applications, such as in molecular switches or sensors where precise control over the transition temperature is essential.

**Figure 4:**
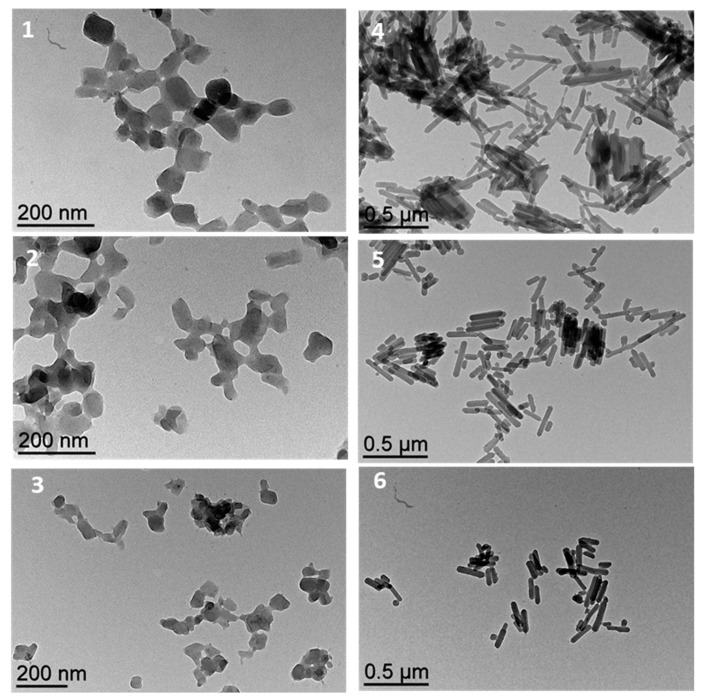
Selected transmission electronic microscopy images for samples **1**–**6**.

Figure 4 presents a TEM image of nanoparticles while varying the amount of TEOS, ranging from 0.25 to 1 equivalent relative to the iron salt, with reaction durations of 15 h and 7 days. The samples demonstrate distinct morphologies contingent on the TEOS quantity and the reaction time.

Samples **1**–**3**, which react during 15 h, exhibit aggregated, rather spherical nanoparticles with a homogeneous shape distribution. A clear decrease in the size of nanoparticles was observed while the quantity of TEOS increased from L = 120 nm with 0.25 eq TEOS to L = 60 nm for 1 eq of TEOS. This tendency could be related to the localization of the TEOS molecules at the interface of reverse micelles, which could act directly on the size of the reverse micelles; the increase in the quantity of TEOS favors smaller-sized micelles and thus smaller nanoparticles.

Samples **4**–**6** show a marked transition in morphology as the reaction time increases. In fact, the prolonged reaction time also contributes to the development of nanorods promoting anisotropic growth. Notably, sample **4** features mainly elongated rods with significant aspect ratios with lengths exceeding 500 nm, although the shapes are irregular. In comparison, samples **5** and **6**, which also contain higher TEOS quantities, produce more regular nanorods with lower aspect ratios. This transformation from spherical nanoparticles to elongated nanorods while increasing the reaction time highlights the critical role of TEOS in determining particle morphology. Moreover, to explain the evolution from a spherical to an anisometric shape, we can imagine that while spherical particles are formed in a first step, like a seed, a prolonged reaction time results in the more stable anisotropic growth, which is certainly in agreement with the intrinsic 1D chain structure’s organization. The microemulsion method, therefore, offers a versatile and robust approach for engineering nanoparticles with tailored properties for advanced technological applications.

**Figure 5:**
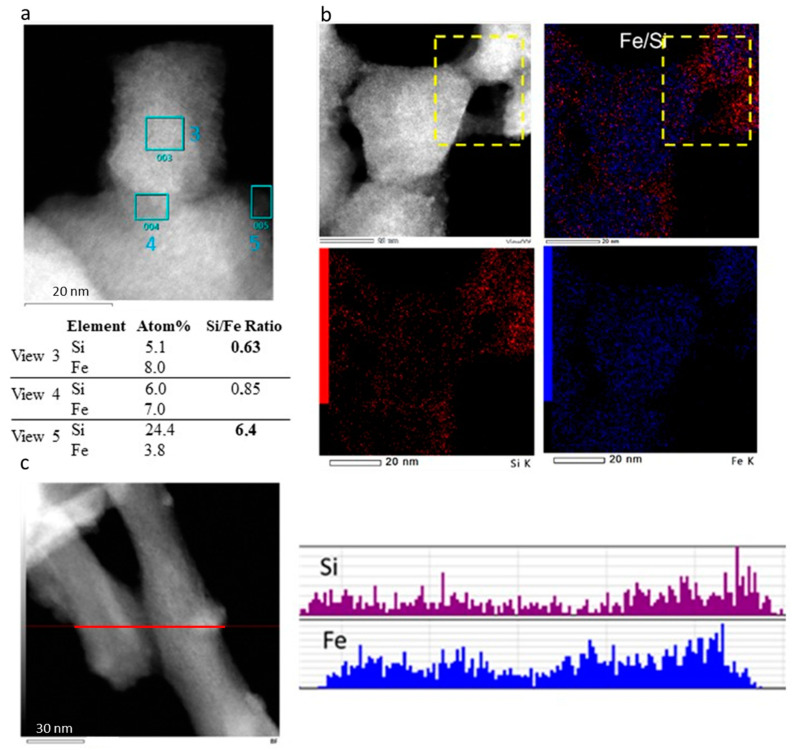
STEM-HAADF images and the corresponding EDX element analyses (**a**), EDX elemental mapping images of overlapped Fe and Si, with the Fe and Si separate (**b**) for sample **2,** and (**c**) STEM-HAADF images of EDX distribution of Fe and Si along a cross-section line for sample **5**.

To characterize the nanohybrid structure of the material, we analyzed the distribution of Si and Fe for sample **2**. EDX analyses revealed a significant increase in the amount of SiO_2_ in the periphery, indicating successful grafting at the surface. Specifically, the EDX results from view 3 showed a Si and Fe ratio of approximately 6.5, while view 1 displayed a ratio of 0.6, as illustrated in Figure 5a. As expected, view 2 shows an intermediate Si and Fe ratio. These data clearly demonstrate that Fe is predominantly located in the core, whereas Si is concentrated at the surface. Furthermore, the composition analysis using EDX elemental mapping confirms this distinct distribution of Fe in the core and Si at the surface. This distribution is particularly evident at the connections between particles, as highlighted in the yellow dash square of Figure 5b. The detailed mapping and analysis underscore the precise assembly and distribution of elements within the complex, providing critical insights into the structural composition of the synthesized nanoparticles.

For the rod-shaped complex **5** obtained from a 7-day reaction, similar conclusions can be drawn. The STEM-HAADF images and line analysis results indicate a distinct distribution of Fe and Si, as depicted in Figure 5c. The analysis shows that Si is more concentrated on the outer surface of the rod, confirming the presence of a partial grafting. This clear demarcation of Si on the exterior underscores the formation of a SiO_2_ grafted structure, with Fe predominantly located in the core and Si forming an outer layer. It is interesting to note that in contrast to the clear core@shell (SCO@SiO_2_) structure obtained for the [Fe(Htrz)_2_(trz)](BF_4_) derivative [38], the partial grafting of the SiO_2_ at the surface of the particles in the present case is in agreement with the evolution of the spherical particles into rod ones with the increase in the reaction time.

## 4. Conclusions

Using a reverse micelles technique to limit and harmonize the growth of the coordination complexes, various sizes and shapes of Fe(NH_2_trz)_3_(BF_4_)_2−x_(SiF_6_)_x/2_@SiO_2_ hybrid particle samples were obtained while modifying both the TEOS concentration and the reaction time. In fact, TEOS, which plays a role in the final morphology of the nanoparticles, also acts as source of the SiF_6_^2−^ counter-anions and SiO_2_ grafting in the hybrid structure. Variation in the SiF_6_^2−^ inclusion at the expense of the BF_4_^−^ leads to a range of mixed-anion compounds exhibiting abrupt spin crossover behavior with hysteresis loop just above room temperature. Thus, in comparison with a previously reported synthesis following conventional coordination chemistry or post synthetic modification (PSM) methods, the reverse micelles technique allows for the control of both the morphology of the particles and the spin crossover properties. Such characteristics associated with the possibility to post-functionalize the SiO_2_ groups make these materials highly promising for integration, notably into polymers, to generate active nanocomposite films and functional devices working at room temperature.

## Figures and Tables

**Table 1:** Experimental conditions, composition, and transition temperatures extracted from reflectivity measurements for all samples (h = hour, d = day).

Sample	T (K)	TEOS	Time	Formulae from ^19^F and ^29^Si NMR	T_1/2_↓	T_1/2_↑
**1**	298 K	0.25 eq	15 h	[Fe(NH_2_trz)_3_](BF_4_)_1.33_(SiF_6_)_0.34_·0.07SiO_2_	303	313
**2**	298 K	0.5 eq	15 h	[Fe(NH_2_trz)_3_](BF_4_)_1.25_(SiF_6_)_0.38_·0.18SiO_2_	310	320
**3**	298 K	1 eq	15 h	[Fe(NH_2_trz)_3_](BF_4_)_1.25_(SiF_6_)_0.38_·0.21SiO_2_	309	320
**4**	298 K	0.25 eq	7 d	[Fe(NH_2_trz)_3_](BF_4_)_1.41_(SiF_6_)_0.30_·0.12SiO_2_	305	315
**5**	298 K	0.5 eq	7 d	[Fe(NH_2_trz)_3_](BF_4_)_1.21_(SiF_6_)_0.39_·0.19SiO_2_	311	320
**6**	298 K	1 eq	7 d	[Fe(NH_2_trz)_3_](BF_4_)_1.16_(SiF_6_)_0.42_	312	325

## Data Availability

The original contributions presented in the study are included in the article/Supplementary Material, further inquiries can be directed to the corresponding author.

## Supplementary Materials

The following supporting information can be downloaded at: https://www.mdpi.com/article/10.3390/nano15020090/s1.
