# Supplementary material for: Various Sizes and Shapes of Mixed-Anion Fe(NH2trz)3(BF4)2−x(SiF6)x/2@SiO2 Nanohybrid Particles Undergoing Spin Crossover Just Above Room Temperature
Source: Nanomaterials (Basel). 2025 Jan 9;15(2):90. doi: 10.3390/nano15020090 (PMC11767441; doi:10.3390/nano15020090)

Various Sizes and Shapes of Mixed-Anion $Fe(NH2trz)_3(BF4)_{2-x}(SiF6)_{x/2}$@$SiO_2$ Nanohybrid Particles Undergoing Spin Crossover Just Above Room Temperature

Xinyu Yang, Rafal Bielas, Vincent Collière, Lionel Salmon * and Azzedine Bousseksou *

Laboratoire de Chimie de Coordination, CNRS & Université de Toulouse (UPS, INP), 31077 Toulouse, France

* Correspondence: lionel.salmon@lcc-toulouse.fr (L.S.); azzedine.bousseksou@lcc-toulouse.fr (A.B.)

Figure S1: Thermogravimetric Analysis (TGA) for sample **1-6**

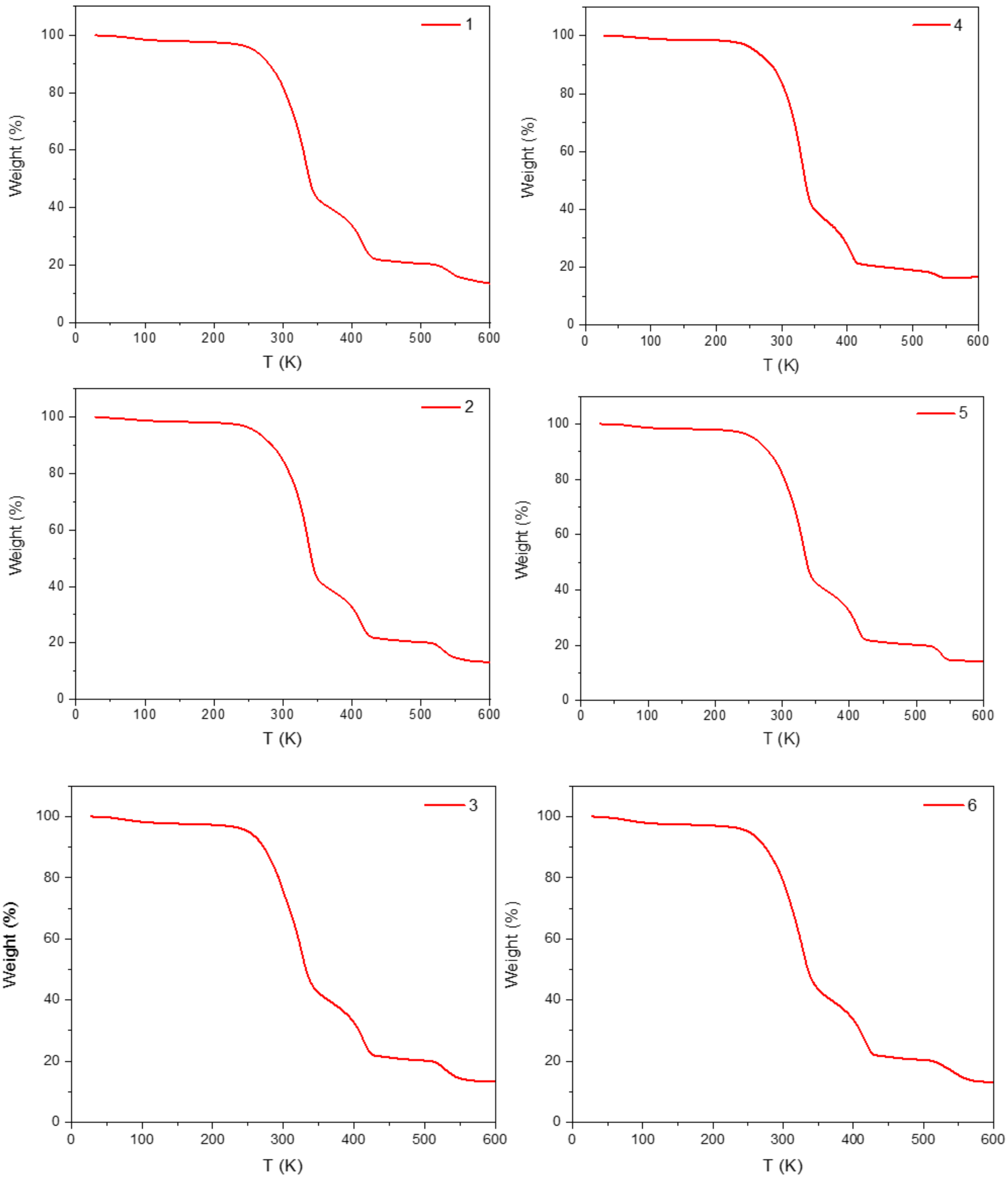

Figure S2: IR spectroscopy for samples **5** and **6**

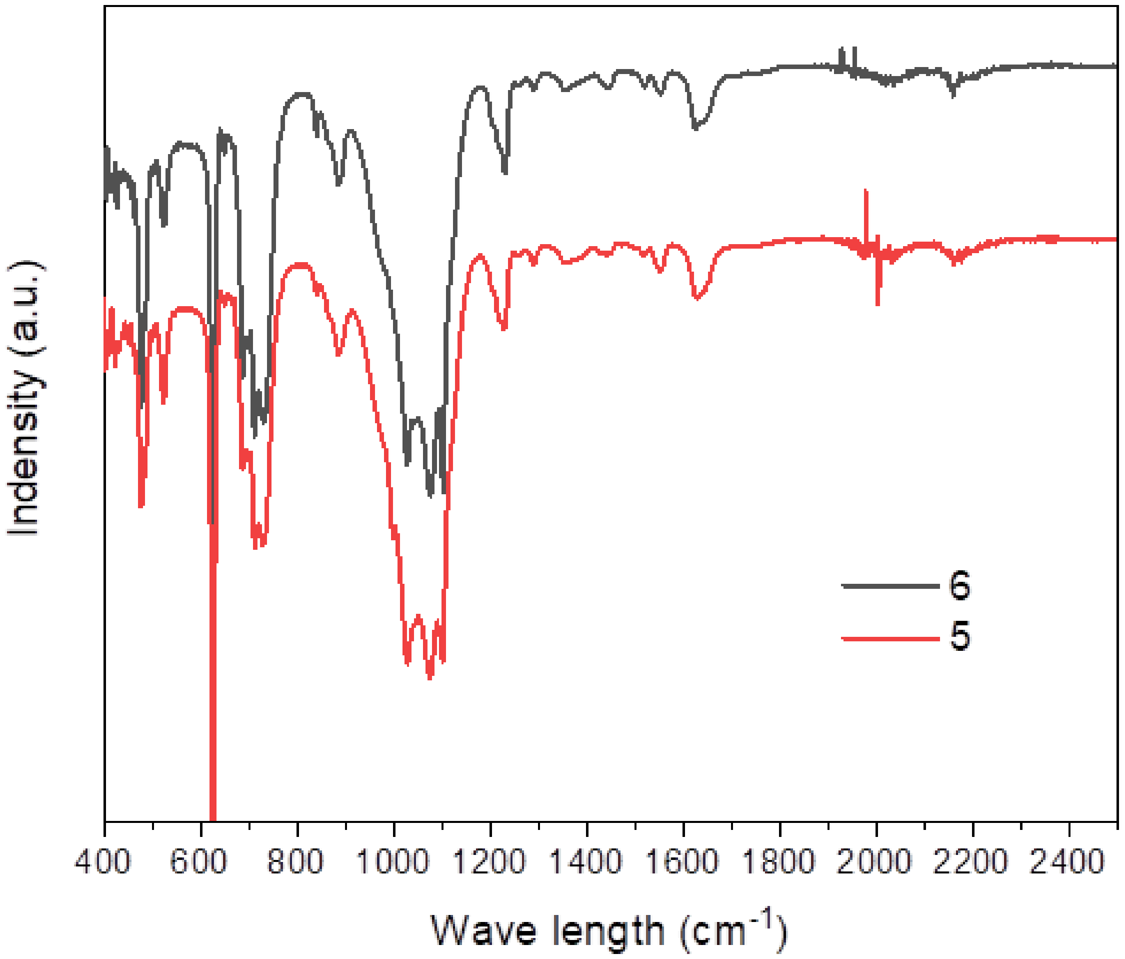

Figure S3: Powder X-ray diffractogram of samples **5** and **6**

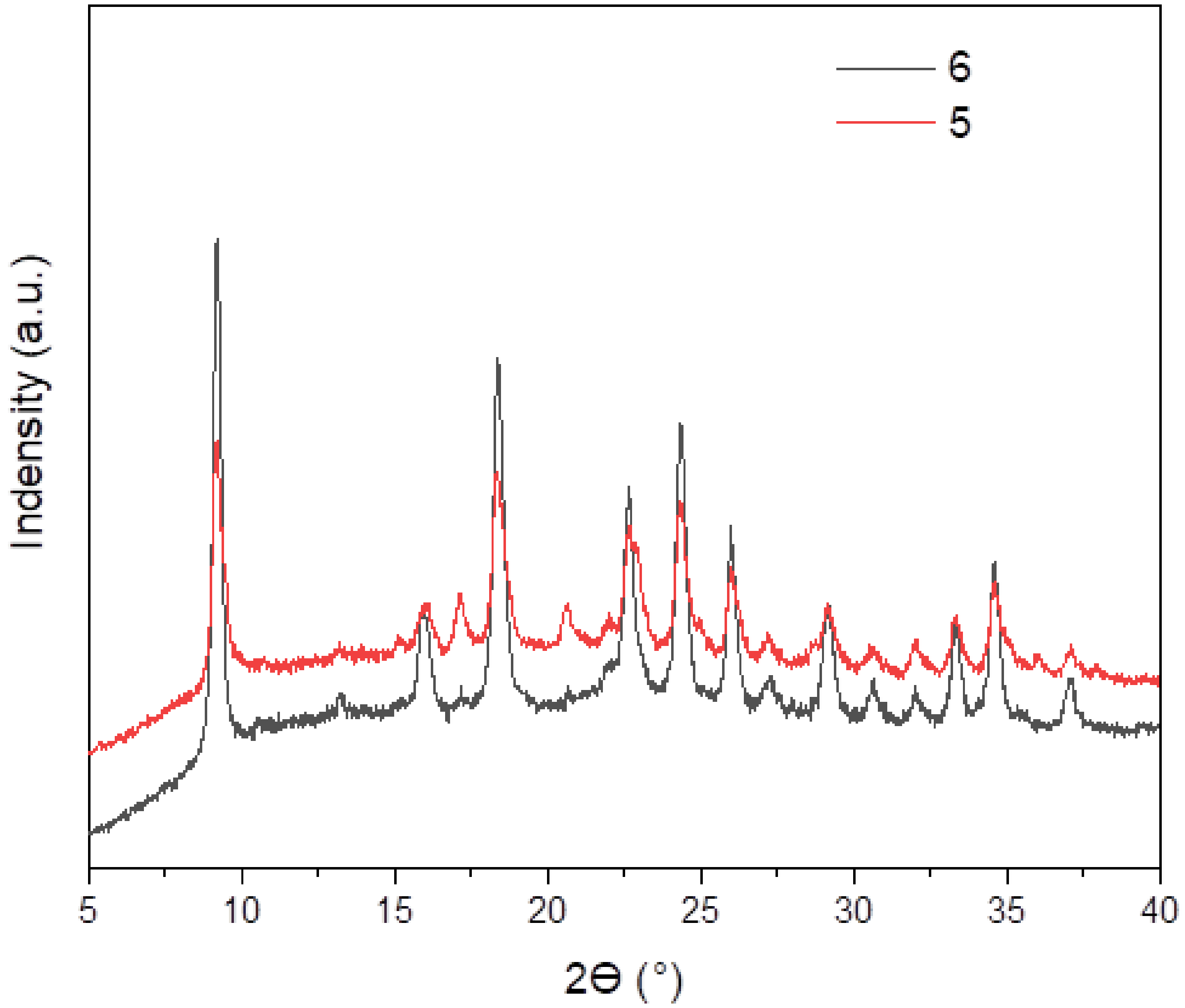

Figure S4: Thermal variation of the optical reflectance for sample **5** (three consecutive thermal cycles)

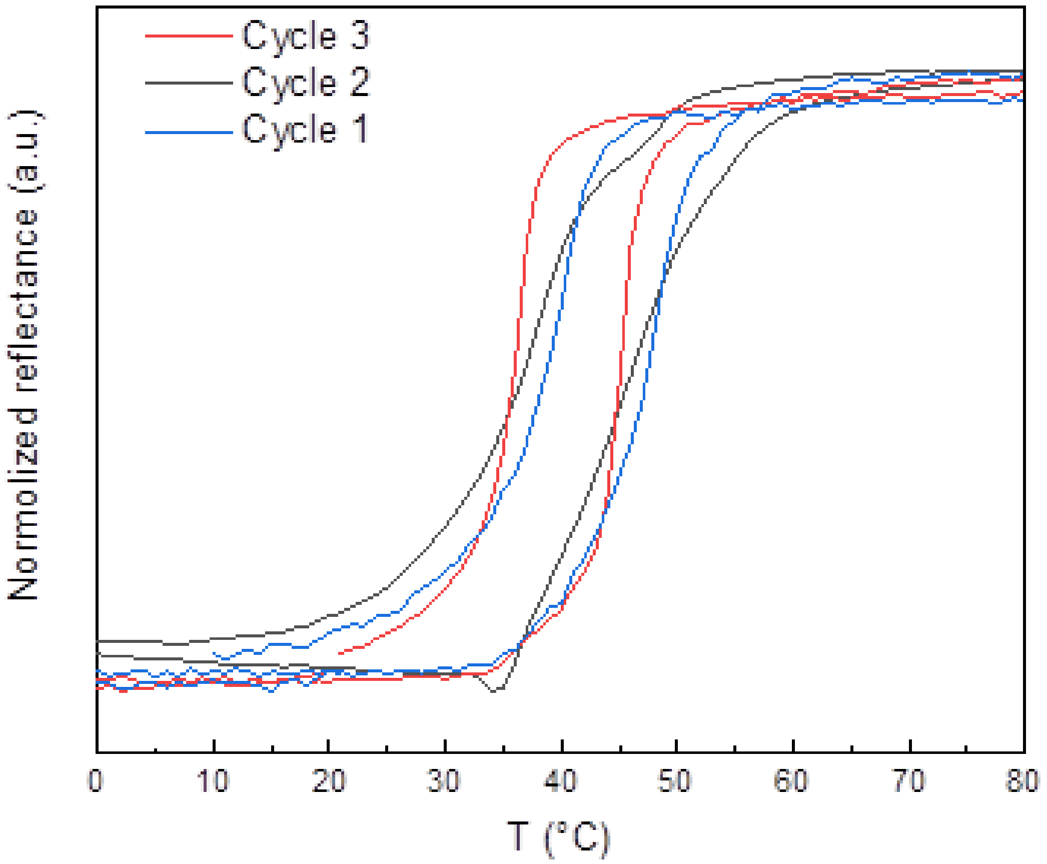

Figure S5: Particle size distributions obtained from TEM images for spherical particles **1-3**

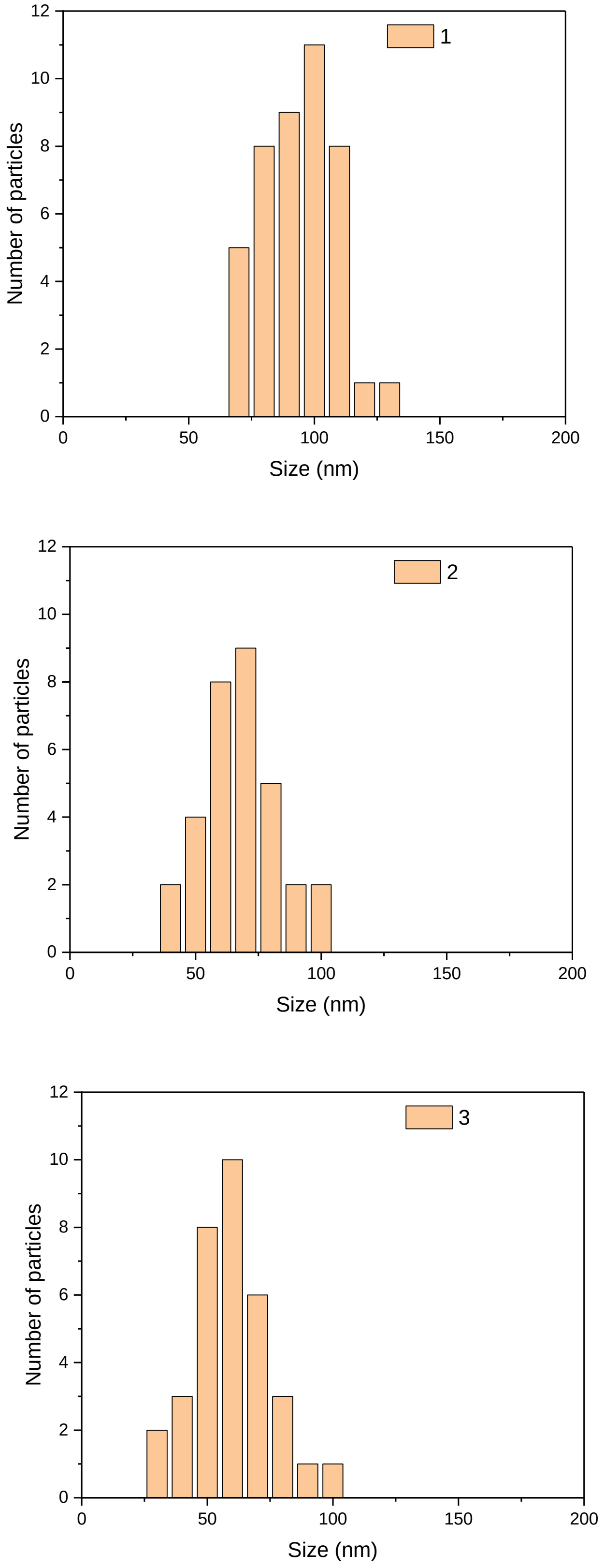

Figure S6: Particle size distributions obtained from TEM images for rod-shaped particles **4-6** (left = length and right = width)

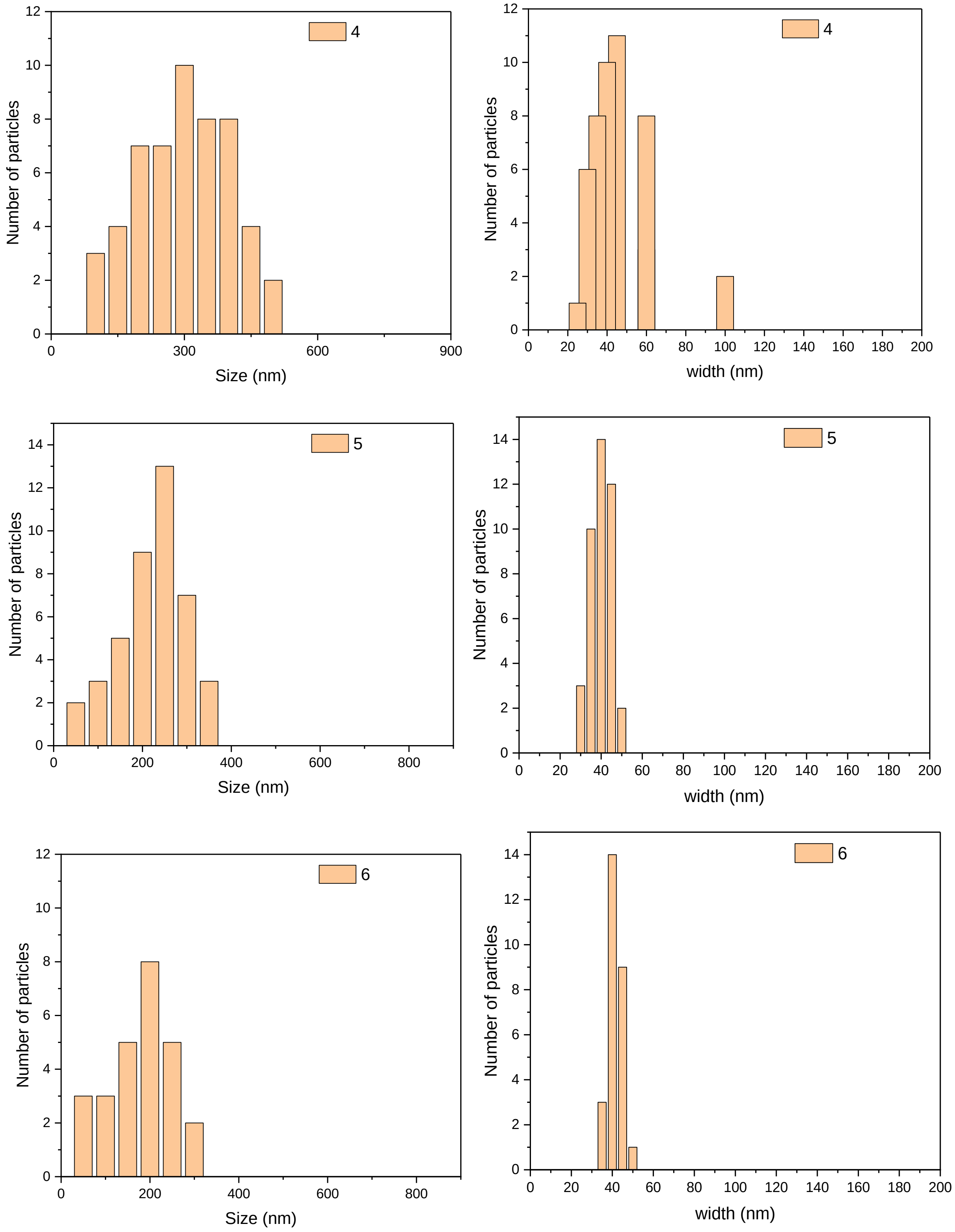

Supplement: Supplementary file 1 [file nanomaterials-15-00090-s001.zip › nanomaterials-3303474-supplementary.pdf]
